# Graphene-Coated PVDF Membranes: Effects of Multi-Scale Rough Structure on Membrane Distillation Performance

**DOI:** 10.3390/membranes12050511

**Published:** 2022-05-10

**Authors:** Emilia Gontarek-Castro, Giuseppe Di Luca, Marek Lieder, Annarosa Gugliuzza

**Affiliations:** 1 Department of Process Engineering and Chemical Technology, Faculty of Chemistry, Gdansk University of Technology, 11/12 G. Narutowicza St., 80-233 Gdansk, Poland; lieder@pg.edu.pl; 2 Research Institute on Membrane Technology, CNR-ITM, Via Pietro Bucci 17/C, 87036 Rende, Italy; g.diluca@itm.cnr.it

**Keywords:** membrane distillation, graphene, multi-scale roughness, antiwetting and antifouling properties

## Abstract

Graphene-coated membranes for membrane distillation have been fabricated by using a wet-filtration approach. Graphene nanoplatelets have been deposited onto PVDF membrane surfaces. Morphology and physicochemical properties have been explored to evaluate the changes in the surface topography and related effects on the membrane performance in water desalination. The membranes have been tested in membrane distillation plants by using mixtures of sodium chloride and humic acid. The multi-scale rough structure of the surface has been envisaged to amplify the wetting and fouling resistance of the graphene-coated membranes so that a better flux and full salt rejection have been achieved in comparison with pristine PVDF. Total salt rejection and an increase of 77% in flux have been observed for coated membrane with optimized graphene content when worked with NaCl 0.6 M (DCMD, ΔT ≈ 24 °C) over a test period of 6 h. The experimental findings suggest these novel graphene-coated membranes as promising materials to develop functional membranes for high-performing water desalination.

## 1. Introduction

In the last century, population and industrial growth have increased producing worldwide demand for water. This request will become even more amplified due to the further demographic and economic expansion along with the persistent exploitation of water basins. The World Water Council estimates that a few billion people living on Earth will suffer from water scarcity or poor water quality by 2030 [1]. Therefore, there is an urgency to identify suitable and eco-sustainable strategies for the management of natural water resources and the production of freshwater. Membrane technology takes advantages over many other technologies due to its easier maintenance, compact modular construction, higher separation factor, no use of chemical additives, and regeneration of spent media [2]. Nowadays, a lot of research is focused on reliable approaches to improve separation performances through the fabrication of novel membranes and/or modification of the existing ones [3,4,5].

Membrane distillation (MD) is a promising and eco-sustainable alternative for seawater desalination [6]. It requires the use of hydrophobic micro-porous membranes that separate feed and permeate streams preventing their mixing. MD is driven by the vapor pressure difference across the membrane, which can be induced by a gradient of temperature or concentration. Numerous hydrophobic membranes have been developed and tested in laboratory on a model brine solution, proving the efficiency of MD in water desalination [7,8,9]. Among them, polypropylene (PP) [10], polytetrafluoroethylene (PTFE) [11] and poly (vinylidene fluoride) (PVDF) [12] are the most popular ones. Nevertheless, membrane fouling and wetting still remain open issues, especially for real seawater processing [13]. For instance, desalination of hypersaline solutions usually leads to scaling at the membrane surface [14], while the treatment of solutions containing organic compounds, including acids and protein-type macromolecules, causes frequently fouling with the subsequent decline of the flux [15]. Due to the interactions between components dissolved in the streams and membrane functional groups, additional layers formation on the membrane surface and pore plugging take place causing increased resistance to the transport [16,17,18,19]. Properly selected membrane materials may reduce the susceptibility to wetting and fouling phenomena [20,21]. Chemical and morphological features can be well addressed in controlling the final performance of membrane separations including membrane distillation [22,23].

It was observed that the creation of a superhydrophobic membrane could effectively deal with wetting and fouling phenomena [7]. There are two main criteria for enhancing membrane hydrophobicity. Membrane surface energy can be reduced and surface roughness can be manipulated [24]. The first criterion is usually achieved using fluoro-polymers or low surface energy modifiers. In the context of surface roughness, it has been observed that the creation of micro- and nanostructured surfaces, also called hierarchical structures, can bring excellent antiwetting properties [25,26]. According to the Cassie–Baxter model [26], rough and complex topography prevent the surface from being penetrated and wetted by the liquid as the air trapped in the grooves between solid substrate protrusions forms an air/solid hydrophobic surface. Due to the increased number of sharp and narrow protrusions for multi-scale rough structures, there is an unusually strong water-repellency on the surfaces, as the contact area between liquid and solid is reduced. A favorable manner to achieve the hierarchical structure is by nanoparticle incorporation. Usually, nanoparticles are incorporated onto a membrane surface by dip-coating method or deposition. For instance, silver nanoparticles have been deposited on PVDF membrane and fabricated membranes showed an increase in the CA and roughness value from 126° up to 136° and from 99.1 to 157.6 nm, respectively [27]. In other studies, polystyrene (PS) microspheres with silica nanoparticles have been coated onto commercial membranes. As fabricated hierarchical membrane exhibited a significant increase in surface roughness value of 343 nm, while the pristine membrane had a roughness of 99 nm [28].

Based on this concept, in this study, a nanostructured coating of graphene nanoplatelets (GNPs) has been fabricated onto the surface of the flat sheet PVDF membrane by using a wet-filtration method. The complex surface topography generated after graphene deposition has been demonstrated to impart much-improved antiwetting and antifouling properties with respect to pristine surfaces. The effectiveness of graphene-coated PVDF membranes has been evaluated during desalination via the direct contact membrane distillation (DCMD) process by working mixtures of NaCl and humic acid (HA) with a degree of salinity of 35 gL^−1^—typical content of salts in seawater. Effects on the flux and rejection as well as recovery of initial permeation properties have been investigated. Based on the results achieved, this kind of graphene-coated membrane exhibits structure-transport properties relationships suitable to enhance their efficiency during the MD process with respect to the non-modified PVDF membranes. The capability of these composite membranes to recover water flux after simple washing cycles with distilled water has been also investigated, resulting in an eco-sustainable cleaning procedure. Thus, the performance of these graphene-coated membranes is promising for the realization of new functional membrane materials to be worked in high-performing MD operations.

## 2. Materials and Methods

### 2.1. Materials

PVDF (Solef^®^6020, Solvay Solexis: water adsorption < 0.040% @23 °C after 24 h; d = 1.78 Kg/m^3^) was donated by Solvay Solexis (Milan, Italy). Hydrophobic graphene nanoplatelets (GPNs) powder with a lateral size > 25 μm, (Sigma-Aldrich, Milan, Italy, Carbon > 95 wt.%; Oxygen < 2 wt.%) was used for membrane coating. N-methyl-2-pirrolidinone (NMP, Riedel de Haëm, Milan, Italy) and 2-propanol (IPA, WWR PROLABO, Milan, Italy) were used for membrane preparation. POREWICK was used for pore size investigation. Sodium chloride (NaCl, WVR PROLABO, Milan, Italy) and Humic acid (HA, Sigma-Aldrich, Milan, Italy) were used for preparing stream solutions.

### 2.2. Preparation of PVDF Membranes

Polymeric solutions were prepared by dissolving 12 wt.% of PVDF powder in NMP under mechanical stirring for 24 h at 30 °C. Then, the solutions were left for 4 h without stirring in order to remove air bubbles. The solutions were cast on a glass support by using a micrometric film applicator (Elcometer) with a gap size of 250 µm. The casting solutions were then coagulated in 2-propanol and washed with deionized water.

### 2.3. WET-Filtration Method

**Figure 1:**
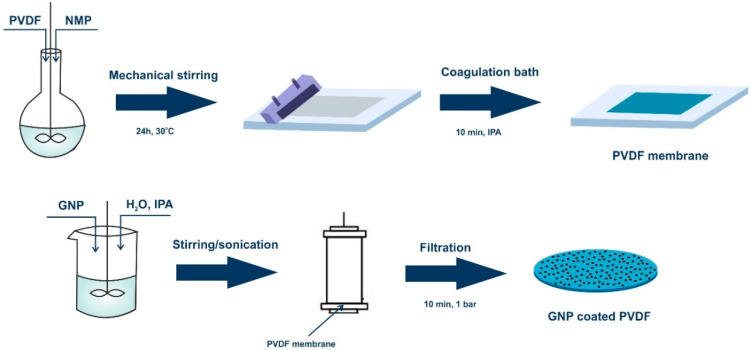
Schematic diagram for the fabrication of the graphene-coated membrane.

Graphene dispersion was prepared by adding graphene to water/2-propanol mixture (volume ratio 7/3) at concentrations of 0.05 mgmL^−1^ and 0.005 mgmL^−1^. Each solution underwent approximately 20 stirring/sonication cycles (20/20 min) to well-disperse the nanomaterials in the solvents. A dead-end filtration cell was used to deposit graphene on the skin layer of PVDF membranes with a diameter of 7 cm. For each dispersion, a volume of 20 mL was poured into the cell and left for 10 min in contact with a membrane surface. Then, a pressure of 1 bar was applied for 10 min in order to remove the mixture of solvents. Each membrane was dried in the air for 24 h. The flat sheet phase inversed PVDF membrane was denoted as pristine PVDF and used for comparison. The coated membranes underwent the leaching test in order to evaluate the stability of attachment of GNPs to the membrane surfaces. A vigorous stirring in distilled water for a period of 24 h was applied for pieces of both coated membranes. Then, the water was filtrated through PTFE filters with 0.2 µm pore size and the filters were subjected to XRD analysis. No graphene traces were found on the filter. A schematic diagram for the fabrication of the graphene-coated membrane is shown in Figure 1.

### 2.4. Membrane Characterization

The morphology of the membranes was analyzed based on the images taken by scanning electron microscope (SEM; Quanta 200, FEI Company, Oberkochen, Germany) and atomic force microscope (AFM, MultiMode 8, NanoScope V, Veeco-Bruker, Plainview, NY, USA). The overall porosity values corresponded to the total void inside the membranes. The pore size was estimated using capillary flow porometer (CFP 1500 AXEL, Porous Materials Inc., Ithaca, NY, USA) [5]. The wetting resistance with pure water and 0.6 M NaCl solution was examined by measuring the contact angle value (Cam 200 KSV instruments, LTD, Helsinki, Finland). Infrared spectra were collected on the top surface of membranes by using a Nicolet iS10 FTIR spectrometer (Thermo Scientific Instrument Co., Boston, MA, USA). X-ray diffractometer ((XRD, Rigaku MiniFlex 600, The Woodlands, TX, USA)) was used to identify the crystalline structure in the range from 5° to 80°.

### 2.5. Membrane Distillation Tests

Thermally-driven MD experiments were performed by using a flat circular module with area of 2.27 cm^2^. The DCMD configuration was chosen and 0.6 M NaCl solutions and related mixtures with HA (0.5 and 1.0 mgmL^−1^) were tested. A flow rate of 2.68 Lh^−1^ at the feed side and 2.23 Lh^−1^ at the permeate side were used. The temperature of feed was kept around 40 °C and a difference of temperature (ΔT) of ~24 °C was applied across the membrane. Each experiment was run for 6 h continuously; retentate and distillate fractions were led in a counter-current flow, toward the membrane cell. The skin side of the membrane was facing the hyper-saline solutions, whereas the reverse side of the membrane was contacting the distillate stream. Two pumps were used to transport the heated feed and cooled permeate to and from the membrane module. The trans-membrane fluxes were estimated by taking into account the weight variations in the distillate with time and the effective surface area of membranes. The salt rejection (R) was determined by measuring the salt concentration on the feed side and permeate side by using a conductivity meter (HI 2300 bench meter supplied by Hanna Instruments, Woonsocket, RI, USA). After MD experiments, the membranes were washed with water at 50 °C for three consecutive cycles, each one of 30 min. Water permeation testing was repeated after working each saline stream.

## 3. Results and Discussion

### 3.1. Chemical and Morphological Features of Membranes

Graphene-coated membranes have been fabricated through a combined phase inversion and wet-filtration approach. Microporous PVDF substrate has been realized according to the immersion precipitation phase inversion and successively has been immobilized by filtration of graphene nanoplates dispersing solutions.

**Figure 2:**
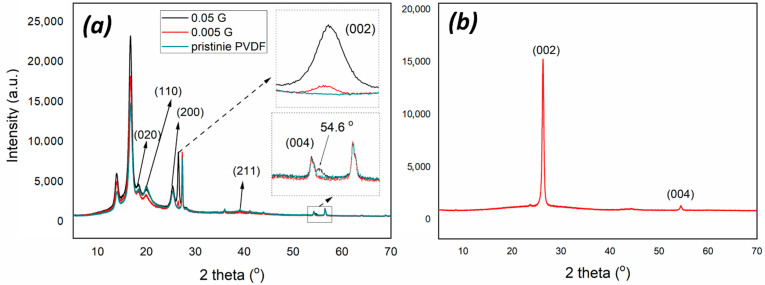
XRD patterns of PVDF and graphene-coated PVDF membranes (**a**), XRD of graphene nanoplatelets (**b**).

**Figure 3:**
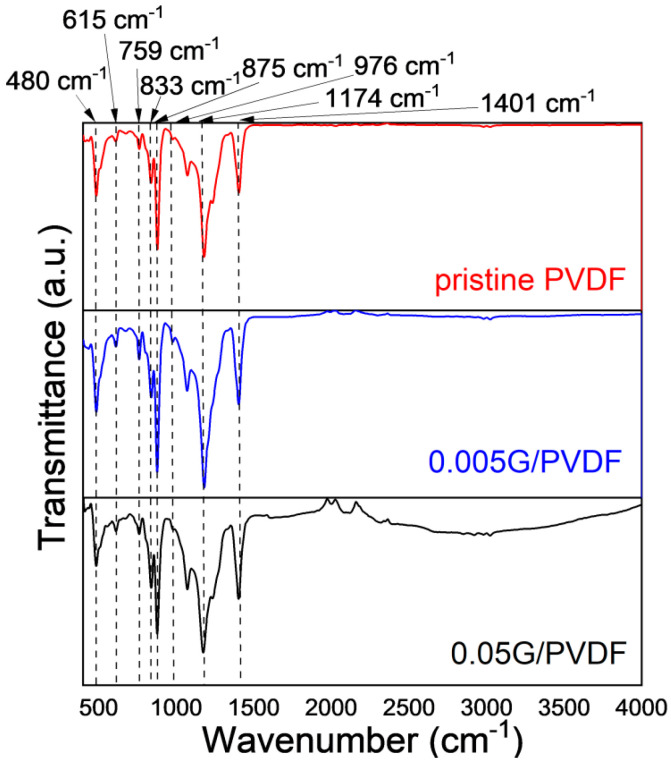
ATR spectra of PVDF and graphene-coated PVDF membranes.

PVDF is a semi-crystalline material and related membranes exhibit a particulate-like morphology while graphene nanoplatelets are dispersed randomly through the membrane surface. XRD crystallographic analyses reveal that PVDF crystalline phase is dominated by both the α and γ phase, as indicated by medium reflections at 18.4 and 20° and weaker at 35.9 corresponding to the reflections of 020, 110, and 200 of monoclinic α-phase crystal as well as weak 39.1 peak corresponding to diffraction peak on planes (211) of monoclinic γ-phase crystal [29] (Figure 2a). The coexistence of two crystalline forms is further confirmed by infrared analysis (Figure 3), showing small peaks at 976 cm^−1^ typical of α-PVDF form, while the band at 833 cm^−1^ is exclusively assigned to the γ-PVDF phase [30]. Further, the crystalline phase of PVDF membranes is dominated by the α phase. XRD spectra collected on 0.05G/PVDF and 0.005G/PVDF membranes show additional peaks at 26.5 (002) and 54.6° (004), which are usually the strongest two in the XRD pattern of natural graphite (Figure 2b). They represent a perpendicular direction (c-axis) to the graphite hexagonal planes, which indicates the presence of graphene on the membranes [31]. FTIR spectra of graphene-coated membranes show additional broad adsorptions in the regions 1500–2000 cm^−1^ and 2500–3720 cm^−1^, especially in the case of the membranes with the largest amount of graphene (0.05G/PVDF). The wide signal centered around 3750 cm^−1^ is associated with hydroxyl groups, whereas the broad region, 1500–2000 cm^−1^ arises from the overlapping of different bands usually falling in the region of 1635–1570 cm^−1^ and assigned to –C=C conjugated stretching and around 1720 cm^−1^ corresponding to C=O stretching vibrations [32]. This yields indication about oxidation and defects of graphene flakes deposited onto the surface.

**Figure 4:**
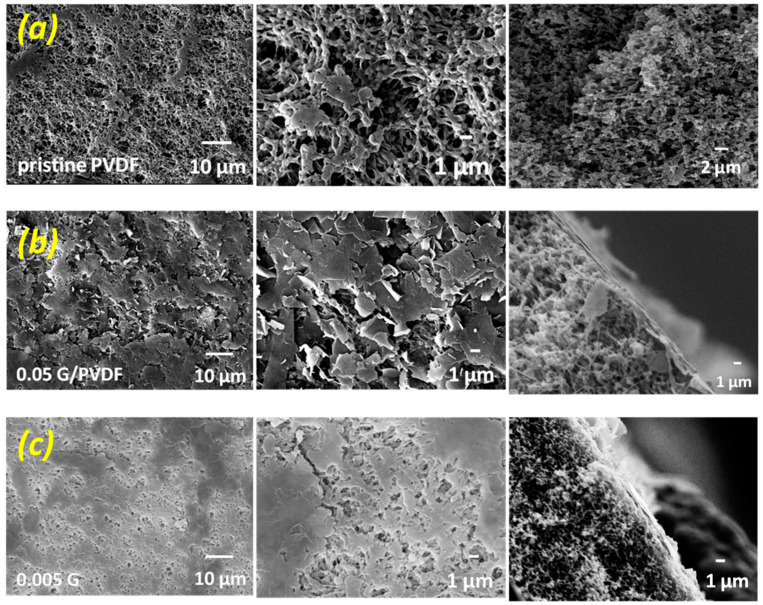
SEM micrographs collected onto membrane surface (first and second column) and across the section (third column) of pristine PVDF (**a**), 0.05G/PVDF (**b**) and 0.005G/PVDF (**c**) membranes.

The crystallization of PVDF polymer chains yields particulate-like morphology during the demixing process leading to the formation of symmetric structures wherein polymer particles are intercalated by voids uniformly distributed through the network (Figure 4a). These free gaps represent the pores of the membranes and determine the overall porosity of the structures, as summarized in Table 1.

PVDF and 0.005G/PVDF membranes show comparable mean and the largest pore size, whereas a reduction is tuned for 0.05G/PVDF. The overall porosity, as well as the thickness, are instead comparable for all membranes. This suggests that the bulk of each membrane is preserved, while graphene platelets are confined to the membrane surface exclusively. SEM micrographs collected across the membrane sections show the formation of a tiny layer onto the top surface of the membranes, especially at higher concentrations of graphene, and confirm that there is no inclusion of flakes inside the membrane structure (Figure 4). The deposition of a larger amount of graphene flakes gives rise to a major obstruction of the free gaps (pores) distributed through the surface, resulting in the formation of a cracked graphene layer mainly (Figure 4b). In the case of lower concentrated graphene dispersion, a random distribution of graphene nanoplatelets is instead observed so that the pores are not clogged, and a large number of free gaps are preserved (Figure 4c).

### 3.2. Wetting Properties of Membranes

**Figure 5:**
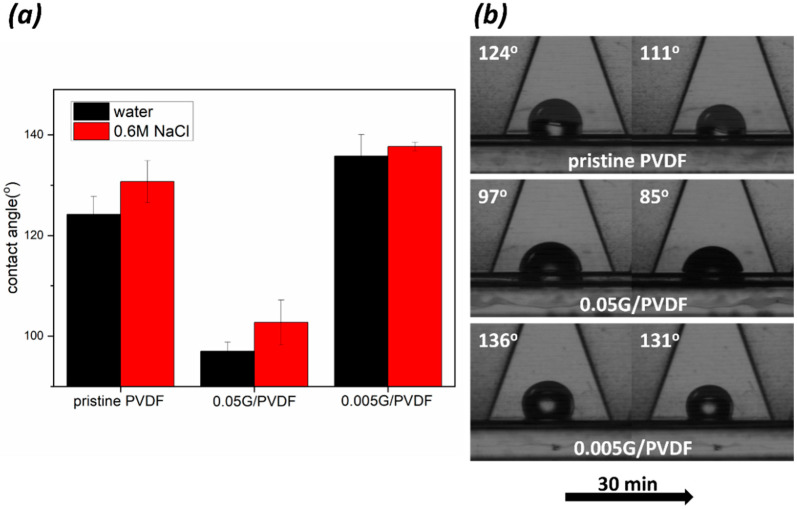
Resistance to wetting of membranes: (**a**) water contact angle of water and 0.6 M NaCl solution at zero time; (**b**) time-dependent images of water droplets within the first 30 min of contact with membrane surfaces.

Chemical and morphological changes in the surface bring inevitably variations in the wetting behavior (Figure 5). A water contact angle value of 124 ± 4° is detected on an unmodified PVDF membrane surface, whereas the deposition of low content of graphene gives rise to the enhanced waterproofness reaching values up to 136 ± 4°. On the contrary, the deposition of a higher concentrated graphene layer causes a significant reduction of hydrophobicity of the surface with contact angle values down to 97 ± 2°. In general, the degree of hydrophobicity of graphene cannot be unequivocally defined, since it is strongly dependent on numerous features such as graphene type, number of layers, defects in the structure, supporting substrate, and impurities, including polymer residues [33,34]. For instance, the study of Wang et al. [35] shows that the water contact angle on a stack of a few graphene layers reached values of 98° while it increases significantly to 127° on a graphene monolayer. On the other hand, Taherian et al. [36], proved that the value of 127° is an unrealistic estimate. They stated that the expected value would be in the range between 95° and 100°. Therefore, the CA estimated for 0.05G/PVDF represents the typical value for the graphene layer. Further, the function of intermolecular interactions established at the graphene and droplet interface and the role of the morphological component of the surface cannot be neglected. In the case of 0.005G/PVDF membranes, the deposition of graphene leads to a non-uniform graphene distribution on the membrane surface with stacked layers that reach thicknesses of 19 ± 5 nm, whilst the surface of 0.05G/PVDF membrane appears to be covered almost entirely with an extent of assembled layers up to 190 ± 24 nm (Figure 2). Consequently, for 0.005G/PVDF membranes effects of both liquid–graphene and liquid–substrate interactions as well as chemical affinity, surface heterogeneities, and surface roughness play a key role in the waterproofness enhancement [37]. The time-dependent images of water droplets within the first 30 min of contact with membrane surfaces confirm improved wetting resistance properties. In this study, it is interesting to observe how water droplet spreads more quickly in the first 30 min when in contact with pristine PVDF and 0.05G/PVDF. A reduction of 10 and 12% can be estimated for the respective contact angle values. Instead, the 0.005G/PVDF membrane continues to exhibit a relatively stable wetting resistance over time with a slight decrease of 4% in the contact angle value.

**Figure 6:**
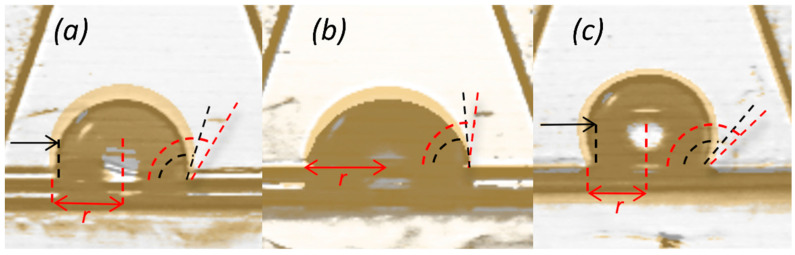
The time-dependent changes in contact radius and contact angle of evaporating droplet on (**a**) pristine PVDF, (**b**) 0.05G/PVDF and (**c**) 0.005G/PVDF surface.

With this regard, it is helpful to consider the slight linear decrease of the droplet size and CA value with time as a consequence of liquid evaporation as well. As known, there are three different evaporation mechanisms that occur in water droplet evaporation on a solid surface [38]. They include a constant contact radius (CCR) configuration with a continuous decrement of the CA values, a constant contact angle (CCA) configuration with a gradual decrease of the solid–liquid interface area (contact radius), and a mixed mode where mixed behavior is observed. The trend of the evaporation rate of water droplets placed over the smooth surfaces depends on the absolute value of CA values; in other words, CCR mode takes place when the CA value is lower than 90° while CCA mode occurs when the CA value tends to be greater than 90°, without considering any effect of surface morphology, as well as the surfaces’ composition [39]. As CCR mode originates from the three-phase contact line pinned to the surface, it is more likely that drop evaporation mode depends strongly on the CA hysteresis than on the absolute CA value [40]. Shin et al. [41] stated that the surface features substantially influence the evaporation rate, and the overall evaporation time takes longer as the membrane surface presents a more hydrophobic nature. In our study, the decrement in the CA value of the tested samples can be observed as the evaporation proceeds. In the case of a 0.05G/PVDF membrane, no contact radius reduction is observed, thus the droplet follows CCR evaporation mode with time, indicating a competitive hydrophilic nature of the surface (Figure 6). Instead, the contact area line of water on pristine PVDF and 0.005G/PVDF recedes, and no pinning is observed. According to Shin et al. [41], there is no pinning (CCR mode) during droplet evaporation on a superhydrophobic surface and the water droplet maintains the spherical shape until the end of evaporation. Our observations prove that 0.005G/PVDF membrane surface shows hydrophobic behavior decisively since the droplet remains in spherical shape—only a slight drop of contact angle is noted—throughout the time of droplet evaporation.

**Figure 7:**
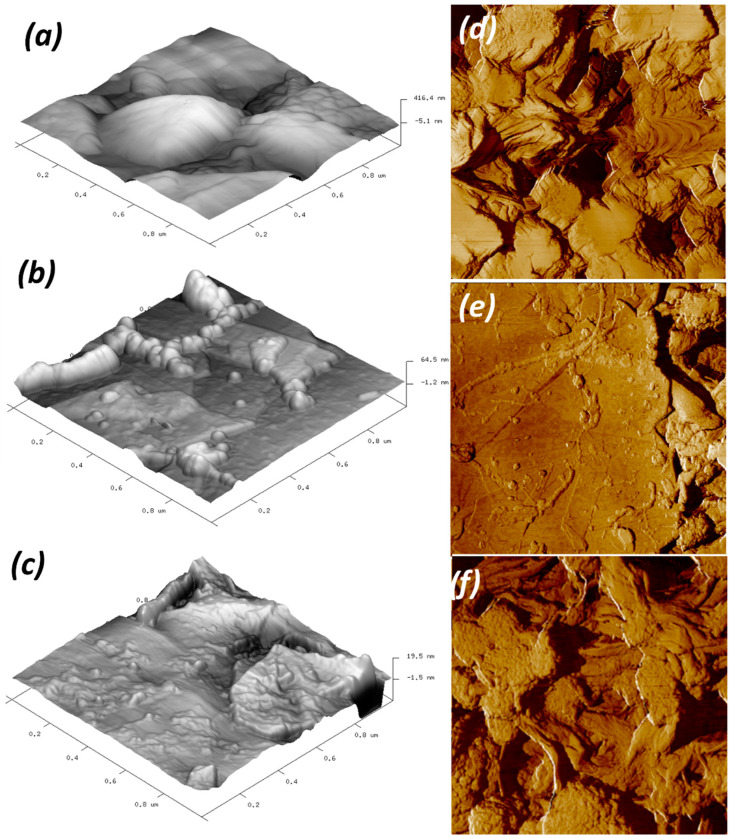
3D AFM images of (**a**) pristine PVDF, (**b**) 0.005G/PVDF, (**c**) 0.05G/PVDF membranes (projected surface 1 µm × 1 µm) and 2D AFM images of (**d**) pristine PVDF, (**e**) 0.005G/PVDF, (**f**) 0.05G/PVDF membranes (projected surface 5 µm × 5 µm).

In the case of 0.05G/PVDF, the slight degree of oxidation could facilitate local graphene–water interactions; however, the dependence of wetting behavior on the nanoscale structure needs to be examined. AFM micrographs reveal that the presence of a low amount of graphene introduces additional nanoscale structures in the surfaces (Figure 7). The significance of multi-scale rough structure on surface hydrophobicity enhancement has been inspired by nature and is already well understood [42]. For instance, the structure of lotus leaves—one of the most famous examples of naturally occurring superhydrophobic surfaces—consists of a combination of micro-roughness (around 10 µm) and nano-roughness (around 100 nm) [43]. The coexistence of these two morphological effects has been demonstrated to enhance antiwetting properties in a membrane coated with fluorinated TiO_2_ or SiO_2_ nanoparticles [44,45]. In our study, AFM analysis indicates an additional fine-structure mainly in graphene-coated membranes obtained from 0.005 mgmL^−1^ graphene liquid dispersions (Figure 7b).

This second topography scale is expected to induce a series of mixed states between Wenzel and Cassie–Baxter with an important contribution to the last one wherein the graphene groove scale is predominant. Values of *Ra* 272 ± 10 nm, *Rq* 327 ± 6 nm, and *Rz* 767 ± 73 have been calculated throughout the 0.005G/PVDF. In a more targeted manner, *Rz* value is the average of the highest peaks and deepest valleys, the PVDF (Table 2). So, the strongest anti-wetting property of 0.005G/PVDF can be plausibly attributed to the multi-scale rough structure.

A larger deposition of graphene flakes produces a new one-scale situation predominantly (Figure 7c), which leads to a more flattened surface with values of *Ra* 97 ± 7 nm, *Rq* 133 ± 15 nm, and *Rz* 180 ± 16 nm. The lowest surface roughness associated with local graphene oxidation is hence expected to produce a lower resistance to the liquid spreading for 0.05G/PVDF membrane surface. Differently from two graphene-coated membranes, the pristine PVDF surface exhibits surface roughness values of *Ra* 214 ± 19 nm, *Rq* 272 ± 10 nm, and *Rz* 499 ± 21 nm, respectively. In this case, Figure 7a shows the presence of pores and other surface deformities due to spherulitic-like polymer particles, which cause a reasonable predominant Wenzel regime with contact angle values of 124 ± 4° and a quicker receding of the contact area line of water (Figure 5 and Figure 6).

It is quite important to observe as NaCl solution spreads less than pure water (Figure 5a). This indicates no interaction between salt, polymer, and graphene in full agreement with the literature. Palacio et al. [46] demonstrated that in NaCl/graphene interface NaCl films adapt to the graphene without disrupting its morphology; no chemical interactions or intercalation takes place with graphene, confirming the total inertness of materials.

### 3.3. Membrane Distillation Experiments

**Figure 8:**
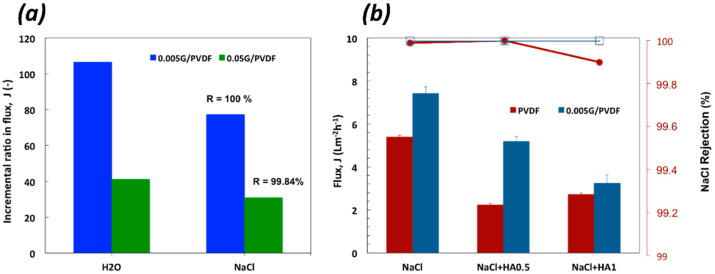
Transport measured through all membranes coming in contact with pure water, NaCl 0.6 M and mixtures of NaCl and HA (0.6 M/0.5 mg mL^−1^ and 0.6 M/1.0 mg mL^−1^): (**a**) Incremental ratio in the flux for 0.005G/PVDF and 0.05G/PVDF membranes with respect to pristine PVDF membrane at T_feed_ = 33 °C; (**b**) Flux (J) and salt rejection (R) estimated at T_feed_ = 40 °C for pristine and 0.005G/PVDF membranes.

DCMD tests have been carried out on pristine PVDF, 0.05G/PVDF, and 0.005G/PVDF membranes. After six running hours, better performance has been tuned for the graphene-coated membranes with an incremental ratio of the flux decisively higher for 0.005G/PVDF as compared to the pristine PVDF membrane (Figure 8a).

An increase of 106 and 77% in flux is measured through 0.005G/PVDF when worked with pure water and NaCl 0.6 M, respectively. Under similar conditions, for 0.05G/PVDF membrane the incremental ratio is of 41% with pure water and 31% with NaCl 0.6 M. A total salt rejection (100%) is obtained with 0.005G/PVDF against a value of 99.84% for 0.05G/PVDF (Figure 8a). The under-performance of 0.05G/PVDF is basically due to smaller pore size (Table 1) and lower resistance to wetting (Figure 5). Subsequently, additional resistance to transport opposes water vapor diffusion while liquid spreading let small saline droplets pass through the membrane pores, reducing slightly the efficiency of the separation. In this context, further experiments have been continued on pristine PVDF and 0.005G/PVDF membranes in order to investigate the antifouling properties and reliability of uncoated and coated membranes. Since humic acid (HA) is considered one of the most significant sources in feed water causing organic fouling, mixtures of NaCl (0.6 M) and HA (0.5 and 1.0 mg mL^−1^) have been processed for 6 h at T_feed_ of 40 °C and under a difference of temperature (ΔT) of ~24 °C across the membranes. Figure 8b shows that already small amounts of HA affect visibly the performance of the pristine PVDF membrane with a reduction of 59% of the flux. A lowering of 30% is instead valued for 0.005G/PVDF at a lower HA concentration (0.5 mg mL^−1^) in the feed. At higher content of HA, an important reduction of the flux is detected for both the membranes, although 0.005G/PVDF continues to exhibit a better performance whatever the operative context examined.

**Figure 9:**
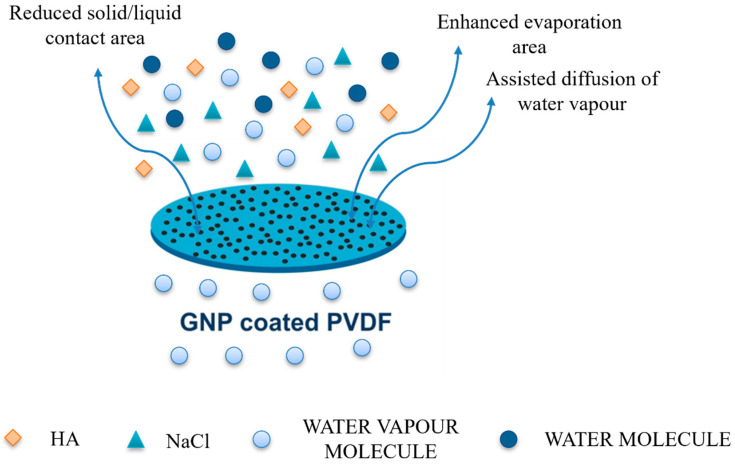
The schematic summarization of enhanced water vapor flux through GNP coated PVDF membranes.

As is well known, there are two possible ways to increase the vapor flux through the membrane during the MD process: (a) to increase the driving force; (b) to reduce the mass transport resistance [7]. The driving force of the process can be increased by enhancing the vapor pressure difference across the membrane. A common approach is to increase the temperature difference between feed and permeate or to increase the effective evaporation area on the feed side. In this study, multi-scale roughness amplifies the mass transfer through the graphene-coated membrane, preventing pore wetting concurrently. The results are higher fluxes along with a total salt rejection (Figure 8b). Indeed, the numerous holes and slots distributed through the graphene-coated surface reduce the contact area between solid and liquid increasing the contact area between membrane and water vapor. As mentioned, 0.005G/PVDF exhibits the highest surface roughness values with the highest standard deviation of the height distribution (*Rq*) and the uppermost average (*Rz*) of the five highest peaks and the deepest valleys along the sampling length (Table 2). Considering that *Rq* is much more sensitive than *Ra* and provides an indication about the nanoscale texture of surface roughness and *Rz* yields indication about skews and kurtosis resulting from the surface roughness profile irregularities, we can assume that the protuberances distributed through 0.005G/PVDF surface cause entrapment of air bubbles. In this way, liquid spreading and intrusion are prevented according to the Cassie–Baxter rules, thereby resulting in a major ability of the membrane to contrast wetting and flooding events. On the other hand, water in a vapor state finds a larger number of free gaps through which it can diffuse. Moreover, considering the defective structure of graphene nanoplatelets, effective sorption sites are available for water vapor molecules, which are, unlike water molecules, devoid of hydrogen bonds. The result is a major interfacial area associated with mass transfer assistance. In our previous study [8], the involvement of graphene nanoplatelets in mass transport during membrane distillation experiments has been demonstrated; the dependence of the mass transport coefficient of water on the amount of graphene confined inside the membranes has been found, thus confirming the occurrence of assisted diffusion of water vapor through the membranes. Therefore, it is not surprising to observe an increase in fluxes when working membrane with graphene flakes onto the surface. The schematic summarization of enhanced water vapor flux is depicted in Figure 9.

**Figure 10:**
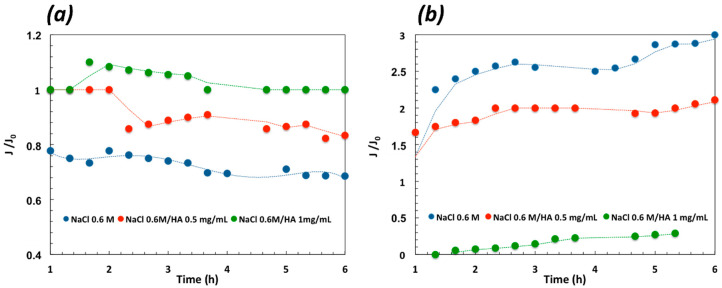
Normalized flux estimated for pristine PVDF (**a**) and 0.005G/PVDF (**b**) when working different NaCl/HA mixtures at T_feed_ 40 °C.

It is also relevant to observe how graphene is actively involved in the mitigation of fouling (Figure 10). The normalized flux is regarded as a good indicator of the capability of membranes to counteract the adhesion of foulants onto the surface. After two running hours, pristine PVDF membranes show a gradual decline in the flux that becomes more pronounced when salt solution is added with HA. On the contrary, the normalized fluxes estimated for the 0.005G/PVDF membrane increase with time, yielding a clear indication of the ability of the graphene nanoplatelets to assist water vapor diffusion through the membranes while aggregations of salts and humic acid onto the surface are contrasted.

Despite that the antifouling properties of graphene are known, nano and micro-scale structures responsible for a multi-level roughness can be further envisaged to prevent wetting inside the membrane pores according to the Cassie–Baxter model. Again, the contact area of water and membrane is reduced as water is supported by a rough surface comprising the protrusions of the membrane and the air pockets between these protrusions. Subsequently, the susceptibility to foulants deposition is expected to decrease differently from what occurs on pristine PVDF membrane surface that is in a predominant Wenzel state [16,47]. In this case, the foulant adsorption takes place more easily leading to an additional resistance to water diffusion.

**Figure 11:**
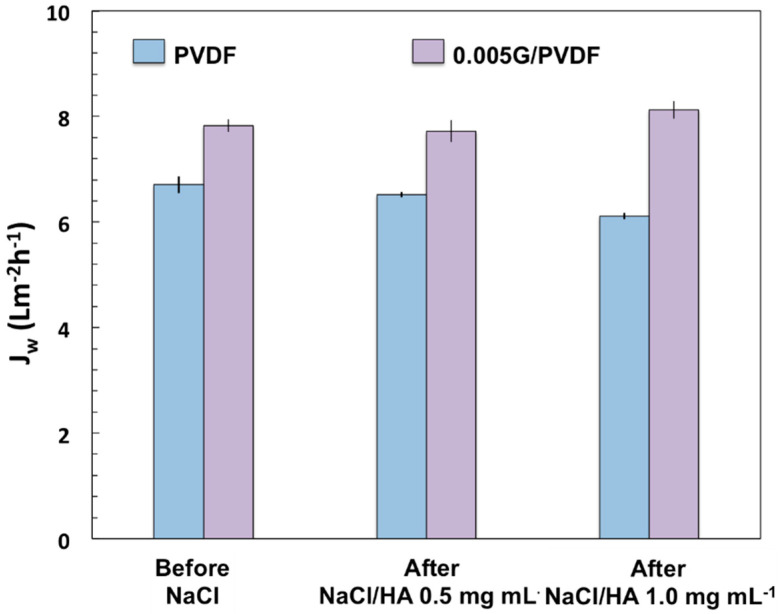
Water flux (J_w_) measured before testing NaCl 0.6 M and after testing NaCl/HA (0.5 and 1.0 mgmL^−1^) solutions at 40 °C (T_feed_).

The fouling is totally reversible for the graphene-coated membrane after three cycles of washing with water at 50 °C (Figure 11). A loss of 9% is instead estimated for pristine PVDF membranes. To sum up, 0.005G/PVDF provides the appropriate roughness without affecting the pore size and porosity of pristine PVDF. Graphene addition generates multi-level roughness on the membrane surface, which leads to waterproofness and enhanced mass transfer and protects against undesired phenomena such as fouling and wetting. As a result, an improved productivity–efficiency trade-off is obtained along with higher durability and chemical resistance.

A comparative analysis reveals as a few graphene nanoplatelets deposited throughout the surface yield the best performance from productivity, selectivity, and economic points of view (Table 3). Although, the examined membranes exhibit a wide range of vapor fluxes (from 3 up to 18 L/m^2^ h), the one produced in this work (0.005G/PVDF) can be considered competitive for the following reasons:

(a) Large flux is obtained at lower feed temperature and using small temperature differences across the membranes, resulting in a cheaper process.
(b) A simpler and inexpensive DCMD configuration can be used to get the best-performing processes for productivity and selectivity under softer working conditions without the necessity to increase electric energy inputs like the VMD configuration.

Most of the works found in the literature report PVDF membranes modified with graphene oxide (GO) or graphene quantum dots (GQDs), while graphene nanoplatelets are not usually employed as additives for surface modification by the wet-filtration approach.

## 4. Conclusions

A new kind of PVDF surface modification by the wet-filtration method has been proposed. Complex topographies due to additional nanoscale structure have been detected on the graphene-coated membranes, especially when low graphene content dispersing solutions have been used. The multilevel surface roughness has been regarded as responsible for a better antiwetting and anti-flooding behavior and an effective evaporation area. MD experiments have been performed using complex mixtures of NaCl and humic acid. Graphene-coated membranes have been demonstrated to have a performance higher than non-modified PVDF and other graphene-functionalized membranes, with a better productivity–rejection trade-off under softer working conditions. After six running hours, the membranes exhibit good stability, mitigated fouling, and total recovery of the initial water permeation. These findings suggest great potential of graphene-coated membranes for highly performing MD processes.

## Figures and Tables

**Table 1:** Structural properties estimated for all membranes.

Sample	Thickness (µm)	Porosity (%)	Largest Pore Size (µm)	Mean Pore Size (µm)
PVDF	50 ± 1	65 ± 4	0.7 ± 0.1	0.48 ± 0.09
0.05G/PVDF	50 ± 1	62 ± 2	0.43 ± 0.09	0.24 ± 0.01
0.005G/PVDF	50 ± 4	63 ± 2	0.80 ± 0.06	0.49 ± 0.01

**Table 2:** Surface roughness estimated for all membranes.

Sample	*Ra* (nm)	*Rq* (nm)	*Rz* (nm)
PVDF	214 ± 19	272 ± 10	499 ± 21
0.05G/PVDF	97 ± 7	133 ± 15	180 ± 16
0.005G/PVDF	272 ± 10	327 ± 6	767 ± 73

**Table 3:** Structural properties estimated for all membranes.

Membrane	MD Configuration	Process Parameters	Flux (L/m^2^ h)	Rejection	Reference
PVDF + GNP (0.5 wt%)	DCMD	0.6 M NaCl Tf: 56 °C Tp: 15 °C	~9	99.9%	[8]
PVDF-f-G	DCMD	0.5 M NaCl Tf: 70 °C Tp: 20 °C	~3	99.9%	[48]
PVDF + GQDs (0.3) wt%	AGMD	0.6 M NaCl Tf: 60 °C Tp: 20 °C	18	99.8%	[49]
PVDF + GO- APTS 0.3%	AGMD	0.6 M NaCl Tf: 85 °C Tp: 23 °C	5.4	99.6%	[50]
PVDF + GO- APTS 0.3%	AGMD	0.6 M NaCl Tf: 85 °C Tp: 25 °C	6.25	99.9%	[50]
Coated 0.005G/PVDF	DCMD	0.6 M NaCl Tf: 40 °C Tp: 16 °C	7.5	100%	This work

## Data Availability

Data is contained within the article.
